# Large-scale in silico identification of drugs exerting sex-specific effects in the heart

**DOI:** 10.1186/s12967-018-1612-6

**Published:** 2018-08-29

**Authors:** Changting Cui, Chuanbo Huang, Kejia Liu, Guoheng Xu, Jichun Yang, Yong Zhou, Yingmei Feng, Georgios Kararigas, Bin Geng, Qinghua Cui

**Affiliations:** 1 Department of Physiology and Pathophysiology, MOE Key Lab of Cardiovascular Sciences, School of Basic Medical Sciences, Peking University, 38 Xueyuan Rd, Beijing, 100191 China; 2 Department of Biomedical Informatics, MOE Key Lab of Cardiovascular Sciences, School of Basic Medical Sciences, Peking University, 38 Xueyuan Rd, Beijing, 100191 China; 3 School of Mathematics Sciences, Huaqiao University, 269 Chenghua North Rd, Quanzhou, 362021 China; 4 Ruike-Donghua Translational Medicine Research Center, Beijing, 100176 China; 5 Department of Cardiology, Beijing Institute of Heart, Lung and Blood Vascular Diseases, Beijing Anzhen Hospital, Capital Medical University, Beijing, 100029 China; 6 Beijing Key Laboratory of Diabetes Prevention and Research, Luhe Hospital, Capital Medical University, Beijing, 101149 China; 7 Department of Endocrinology, Luhe Hospital, Capital Medical University, Beijing, 101149 China; 8 Berlin Institute of Health, Institute of Gender in Medicine and Center for Cardiovascular Research, Charité-Universitätsmedizin Berlin, Berlin, Germany; 9 DZHK (German Centre for Cardiovascular Research), partner site Berlin, Berlin, Germany; 10 Hypertension Center, Fuwai Hospital, Chinese Academy of Medical Sciences and Peking Union Medical College, State Key Laboratory of Cardiovascular Disease, National Center for Cardiovascular Diseases, Beijing, 100037 China; 11 Center of Bioinformatics, Key Laboratory for Neuro-Information of Ministry of Education, School of Life Science and Technology, Chengdu, 610054 China

**Keywords:** Sex differences, Cardiovascular drug, Precision medicine

## Abstract

**Background:**

Major differences exist between men and women in both physiology and pathophysiology. Dissecting the underlying processes and contributing mechanisms of sex differences in health and disease represents a crucial step towards precision medicine. Considering the significant differences between men and women in the response to pharmacotherapies, our aim was to develop an in silico model able to predict sex-specific drug responses in a large-scale.

**Methods:**

For this purpose, we focused on cardiovascular effects because of their high morbidity and mortality. Our model predicted several drugs (including acebutolol and tacrine) with significant differences in the heart between men and women. To validate the sex-specific drug responses identified by our model, acebutolol was selected to lower blood pressure in spontaneous hypertensive rats (SHR), tacrine was used to assess cardiac injury in mice and metformin as control for a non-sex-specific response.

**Results:**

As our model predicted, acebutolol exhibited a stronger decrease in heart rate and blood pressure in female than male SHRs. Tacrine lowered heart rate in male but not in female mice, induced higher plasma cTNI level and increased cardiac superoxide (DHE staining) generation in female than male mice, indicating stronger cardiac toxicity in female than male mice. To validate our model in humans, we employed two Chinese cohorts, which showed that among patients taking a beta-receptor blocker (metoprolol), women reached significantly lower diastolic blood pressure than men.

**Conclusions:**

We conclude that our in silico model could be translated into clinical practice to predict sex-specific drug responses, thereby contributing towards a more appropriate medical care for both men and women.

**Electronic supplementary material:**

The online version of this article (10.1186/s12967-018-1612-6) contains supplementary material, which is available to authorized users.

## Background

There are significant differences between men and women in physiology, in the development and outcome of disease, as well as in the response to pharmacotherapies [1–3]. However, the role and effects of biological sex have been poorly investigated on the basis of the assumption that men and women respond similarly to disease and therapeutic interventions [3, 4]. Dissecting the differences between male and female individuals in health and disease is being recognized as critically important for improving the efficiency, efficacy, accuracy and precision of medical care [3, 5].

Among others, genetic mechanisms are expected to contribute to sex differences in physiology, pathophysiology and drug responses [3, 6]. In the post-genomic era, the fast development of high-throughput techniques has enabled the identification of a large number of genes that are under sex-specific regulation in several tissues [7]. For example, using transcriptomic approaches, projects, such as the Genotype-Tissue Expression (GTEx), have identified hundreds of genes that are differentially expressed between the sexes in various tissues [8, 9]. These studies have made important contributions towards the understanding of the biology of sex differences, but methods to infer sex-specific medicine based on functional genomic datasets are still limited.

Public databases [10, 11] have accumulated enormous profusion of functional genomic datasets, which may facilitate the research and development of novel and innovative in silico models for the prediction of sex-specific drug responses. On the basis of this, we propose here an in silico model, SexBiasedDrug, which infers drugs that exert different effects between male and female individuals. For this purpose, we focused on cardiovascular effects because of their high morbidity and mortality, based on acquired big functional genomic data responses to many drugs. SexBiasedDrug first calculates the male-biased genes (MGs) and female-biased genes (FGs) based on gene expression profiles from the public GEO database [10] and then determines the Connectivity Map [11] drugs that show significant associations to MGs and FGs. These resulting drugs are the ones exerting sex-specific effects. Finally, we confirmed the accuracy of our model using experimental animals treated with acebutolol, tacrine and metformin and show its clinical relevance employing two Chinese cohorts.

## Methods

### Generation of the in silico model

We used the normalized human cardiac gene expression profiles (GEO Accession Number: GSE57345-GPL11532 [12]) from the GEO database [10]. In the original study, Liu et al., studied the changes in cardiac gene expression between failing and non-failing hearts. We previously presented a method to identify sex-biased genes from public gene expression datasets [13]. Here, we used this approach to identify sex-biased genes in the healthy heart. For this purpose, we only extracted the 135 gene expression profiles from non-failing (normal control) heart samples and classified them into two groups (male group and female group) based on the sex annotation. There was no difference in the age between the male and female groups (data not shown, Wilcoxon test). Next, we compared the expression level between the two groups for each gene using multiple *t* tests. The male/female fold change was calculated. The *P* values were corrected by FDR. We considered genes with FDR ≤ 0.05 to be sex-biased.

We used gene expression signatures following treatment with more than 1000 drugs from the Connectivity Map [11], which is a database with thousands of gene expression profiles for various drug treatments and was originally used for drug-repurposing. Here, we used the data in the Connectivity Map for predicting sex-biased responsive drugs. For a given drug treatment, genes with fold change ≥ 2.0 and ≤ − 2.0 between treatment and control were considered upregulated and downregulated, respectively. Next, we evaluated the association of sex-biased genes and a given drug by the Fisher’s exact test, which was performed based on two kinds of classifications, i.e. sex-biased gene and drug-regulated gene. Additional file 1 lists the top 20 drugs that show the most significant sex-specific effects in the heart. We developed a computer script using R and Java, SexBiasedDrug, for this purpose. Finally, it should be noted that a bigger/smaller fold change does not always mean more/less significant. Therefore, methods to identify better gene signatures could be proposed in the future.

### Animal experiments

All animal procedures complied with the Animal Management Rule of the Ministry of Health, People’s Republic of China (Document No. 55, 2001) and the Care and Use of Laboratory Animals published by the US National Institutes of Health (NIH Publication No. 85-23, updated 2011). The care and use of laboratory animals was approved by the Laboratory Animal Ethics Committee of Peking University. Male and female Sprague–Dawley (SD) rats (200–220 g), Spontaneously Hypertensive Rats (SHR) (9 weeks old), C57BL/6J mice (20–25 g) were purchased from the Animal Center of Peking University Health Science Center. Animals were kept under temperature 23 ± 2 °C, humidity range of 40–70% and 12-h light/dark cycles. Food and water were given ad libitum. Acebutolol hydrochloride, isoproterenol, tacrine, metformin, and dihydroethidium (DHE) were obtained from Sigma-Aldrich. Other chemicals and reagents were of analytical grade.

For the animal experiments with acebutolol, eight male and eight female SHRs were treated with acebutolol (50 mg/kg/day, gavage), control groups were given gavage of equal volume saline. The changes of blood pressure were recorded via a noninvasive tail-cuff apparatus (Softron, Japan). Blood pressure was calculated as the average value of three successive measurements. Animals were anesthetized with isoflurane and then pentobarbital sodium (50 mg/kg) was given by intraperitoneal injection. Heart rate changes were recorded by electrocardiogram (ECG) transduction sensor connecting with Powerlab 4S (Adinstrument, Australia).

For tacrine and metformin treatments, 10 male and 10 female C57BL/6J mice were administered tacrine (5 mg/kg/day, gavage) for 4 weeks; or treated with metformin (30 mg/kg/day, gavage). Control mice were given equal volume of saline. Blood samples were collected from the angular artery. Serum cardiac troponin I (cTNI) was measured by an ELISA kit (R&D System Inc.) according to the manufacturer’s protocol. Mice were sacrificed and the heart was quickly removed. Frozen cardiac sections (6 μm) were stained with dihydroethidium (DHE, 5 μM) at room temperature for 60 min and images were acquired with a fluorescence microscope (90i, Nikon, Japan).

### Study population

Consecutive patients were recruited at two Chinese sites. One diabetes cohort was obtained from Luhe Hospital at Beijing city with 35 patients receiving metoprolol (9 men and 26 women). The other cohort was from the Jidong community for health management at Tangshan city with 153 patients (86 men and 67 women) receiving metoprolol. The basal clinical characteristics are shown in Tables 1 and 2, respectively. The investigation conforms with the principles outlined in the Declaration of Helsinki and patient written informed consent was obtained.

**Table 1:** The clinical characteristics of diabetes cohort from Beijing

	Men	Women	P values
*N*	9	26	
Age	55.11 ± 6.51	55.15 ± 6.27	0.9865
SBP (mmHg)	139.41 ± 14.30	131.05 ± 13.95	0.1447
DBP (mmHg)	89.11 ± 8.69	77.19 ± 8.21	0.0011
HR (bmp)	75 ± 12	75 ± 9	0.9920
BMI (kg/m^2^)	24.63 ± 2.58	28.20 ± 3.56	0.0112
Glu (mM)	4.95 ± 0.58	5.23 ± 0.44	0.1487
TG (mM)	1.46 ± 1.16	1.92 ± 0.82	0.2187
TC (mM)	4.87 ± 0.38	5.03 ± .81	0.5711
HDL-c (mM)	1.35 ± 0.38	1.24 ± 0.23	0.2967
LDL-c (mM)	2.92 ± 0.31	3.11 ± 0.71	0.4584
Other medication	ACEI (0)	ACEI (0)	
	ARB (0)	ARB (2)	
	CCB (1)	CCB (4)	
	Diuretics (1)	Diuretics (1)	
Co-morbidities	Diabetes (9)	Diabetes (26)	
	CHD (1)	CHD (1)	

*SBP* systolic blood pressure, *DBP* diastolic blood pressure, *HR* heart rate, *BMI* body mass index, *Glu* fasting serum glucose, *TG* serum triglyceride, *TC* serum total cholesterol, *HDL* high density lipoprotein cholesterol, *LDL-c* low density lipoprotein cholesterol, *ACEI* angiotensin converting enzyme inhibitors, *ARB* angiotensin receptor blockers, *CCB* calcium channel blockers, *CHD* coronary heart disease

**Table 2:** The clinical characteristics of health management cohort from Tangshan

	Men	Women	P values
*N*	86	67	
Age	55.23 ± 10.56	59.55 ± 7.65	0.0039
SBP (mmHg)	149.2 ± 16.45	153.3 ± 20.84	0.1809
DBP (mmHg)	96.63 ± 11.44	89.27 ± 13.43	0.0004
HR (bmp)	72.66 ± 11.61	72.48 ± 14.07	0.9362
BMI (kg/m^2^)	26.78 ± 2.99	26.42 ± 3.52	0.4875
Glu (mM)	6.21 ± 2.04	6.11 ± 2.02	0.7596
TG (mM)	1.93 ± 1.29	2.29 ± 1.72	0.1581
TC (mM)	4.74 ± 1.01	5.17 ± 0.87	0.0067
HDL-c (mM)	1.08 ± 0.18	1.21 ± 0.28	0.0015
LDL-c (mM)	2.74 ± 0.65	2.93 ± 0.60	0.0599
Other medication	ACEI (1)	ACEI (4)	
	ARB (3)	ARB (1)	
	CCB (11)	CCB (5)	
	Diuretics (3)	Diuretics (1)	
Co-morbidities	Diabetes (29)	Diabetes (15)	
	Stroke (6)	CHD (3)	

*SBP* systolic blood pressure, *DBP* diastolic blood pressure, *HR* heart rate, *BMI* body mass index, *Glu* fasting serum glucose, *TG* serum triglyceride, *TC* serum total cholesterol, *HDL* high density lipoprotein cholesterol, *LDL-c* low density lipoprotein cholesterol, *ACEI* angiotensin converting enzyme inhibitors, *ARB* angiotensin receptor blockers, *CCB* calcium channel blockers, *CHD* coronary heart disease

### Statistical analysis

All data are expressed as mean ± SD. Differences among groups were analyzed by two-way ANOVA (multiple groups) or two-tailed *t* test (two groups). The significance level was set at 0.05.

## Results

### An in silico model for inferring drugs that exert sex-specific effects

**Fig. 1:**
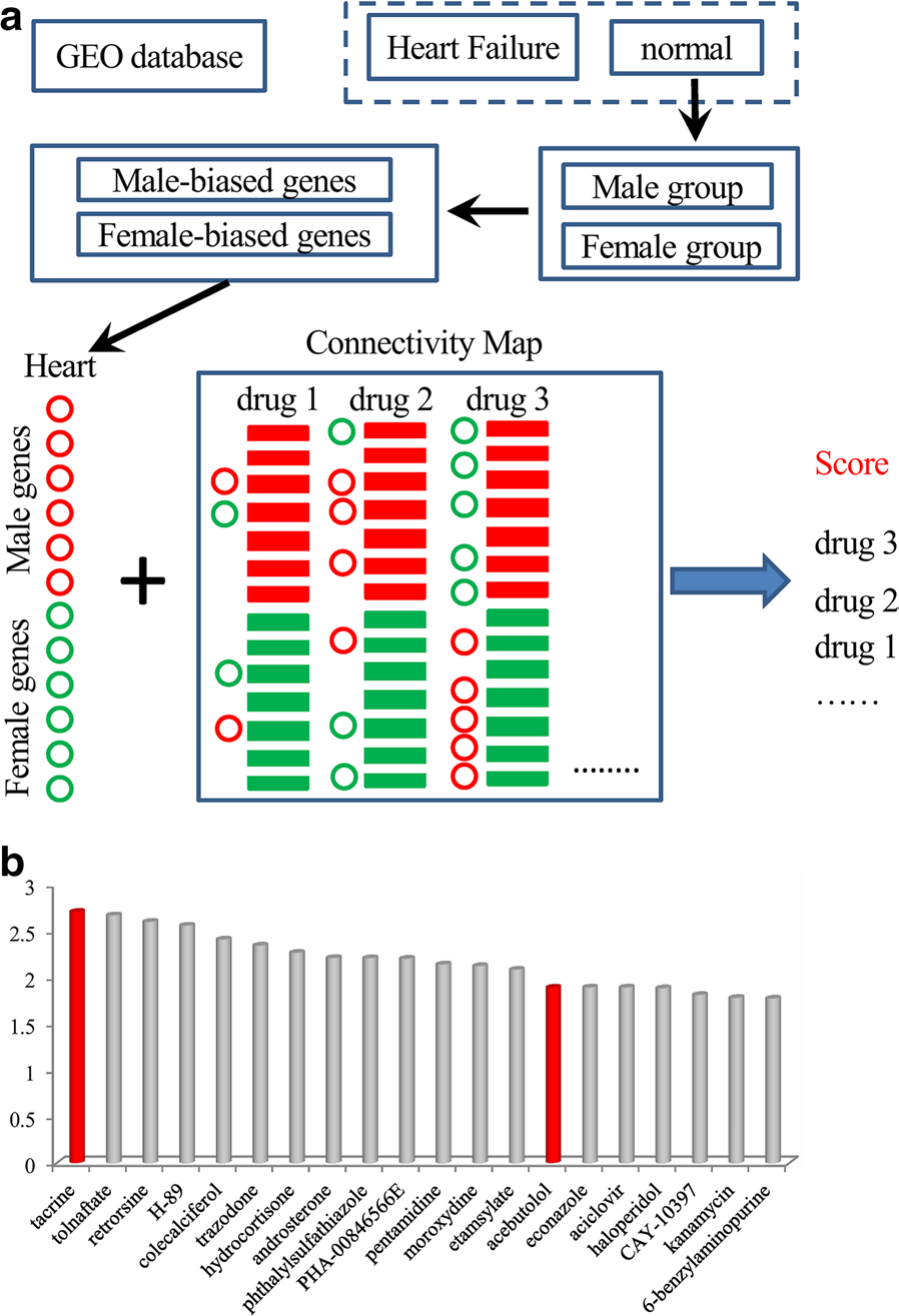
The framework for an in silico prediction model of sex-biased drug responses. The in silico pipeline first identified sex-biased genes in healthy cardiac tissues from a GEO public gene expression dataset (**a**). Then the pipeline build associations between sex-biased genes from the GEO dataset and drug-deregulated genes from the Connectivity Map database. The drugs with significant associations with the heart sex-biased genes will be predicted to have sex-biased effects on human heart. Top 20 predictions (**b**)

We have developed an in silico pipeline, SexBiasedDrug, which predicts drugs exerting different effects between male and female individuals. For this purpose, we focused on public datasets (GEO: GSE57345-GPL11532) [12] of non-failing and failing human hearts (Fig. 1a). We analyzed 135 gene expression profiles from non-failing cardiac samples for sex-specific gene expression (72 male vs. 63 female samples). There was no significant age difference between the groups (data not shown). We identified 134 sex-biased genes (Additional file 2); 79 male-biased genes (MGs, which are higher in males than in females) and 55 female-biased genes (FGs, which are higher in females than in males). We next determined the associations between the sex-biased genes (MGs and FGs) and the deregulated genes (upregulated genes, UGs, and downregulated genes, DGs) induced by drugs in the Connectivity Map database one by one using Fisher’s exact test (Fig. 1a). Additional file 1 shows the top 20 drugs that are predicted to exert significantly different effects between men and women (Fig. 1b).

### Experimental validation of the in silico model

**Fig. 2:**
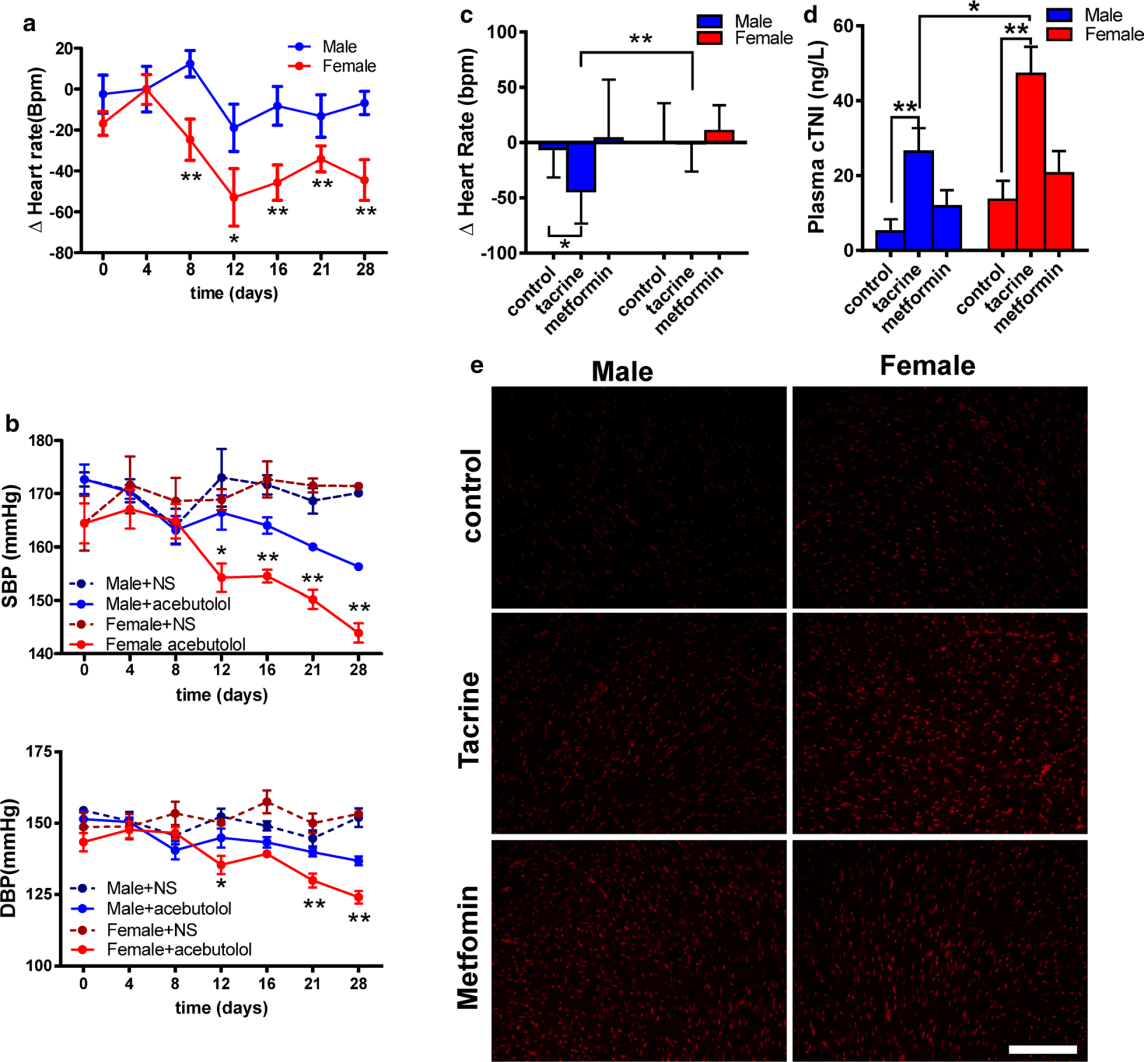
Sexual differences of therapeutic evaluation for acebutolol and tacrine on heart. **a** Acute administration acebutolol, the change of heart rate was assessed. Treatment SHR with acebutolol for 4 weeks, the changes of heart rate (**b**) and blood pressure (**c**) were determined. Administration tacrine for 3 weeks, the heart rate changes (**d**), plasma cTNI level (**e**) and superoxide generation of heart by DHE staining were measured in mice, and metformin as a negative control for sexual differences. All data are present as mean ± SD. *P < 0.05; **P < 0.01 between two groups

To confirm the accuracy of our in silico pipeline, SexBiasedDrug, we selected two positive predictions for sex-specific effects, i.e. acebutolol and tacrine, and one negative prediction for sex-specific effects, i.e. metformin. Acebutolol is a selective β1 receptor blocker used mainly for the treatment of hypertension and arrhythmias [14]. As our in silico model predicted, following a 4-week treatment of spontaneously hypertensive rats (SHR) with acebutolol, the heart rate was significantly lower in female than male SHRs (Fig. 2a). Similarly, systolic blood pressure (SBP) and diastolic blood pressure (DBP) were significantly lower in female than male SHR treated with acebutolol (Fig. 2b). These data together confirmed our in silico prediction that acebutolol exerts sex-specific effects.

Tacrine is a cholinesterase inhibitor, which was aimed for the treatment of Alzheimer’s disease but has been discontinued from the US market [14]. Given that tacrine could exert cardiac-toxic effects leading to bradycardia, we investigated heart rate and cardiac toxicity after tacrine treatment, using metformin as negative control for the prediction of sex-specific effects. In agreement with our in silico predictions, after 3 weeks of treatment of healthy mice, tacrine significantly decreased heart rate in male mice but not in female mice (Fig. 2c). In contrast, metformin did not affect the heart rate in both male and female mice (Fig. 2c). We further found that plasma cardiac troponin I (cTNI) levels (Fig. 2d) and cardiomyocyte superoxide generation, as assessed by dihydroethidium (DHE) staining (Fig. 2e), were induced to a significantly higher extent in female than male mice (Additional file 3: Figure S1). Metformin also slightly increased cardiomyocyte superoxide level, but there was no significant difference between male and female animals (Fig. 2e, Additional file 3: Figure S1). The changes in cTNI levels and superoxide generation indicate that tacrine exerted more severe toxicity in female than male mice. These data together further support the accuracy of our in silico model for the prediction of sex-specific drug effects.

### Clinical validation of the in silico model

To demonstrate the clinical relevance of our model, we performed a retrospective study comparing the blood pressure changes between male and female patients receiving metoprolol in two Chinese communities. In China, acebutolol had been little used because of its side-effects and it was replaced with metoprolol. The chemical structure of metoprolol is close to that of acebutolol in chemical structure (Additional file 4: Figure S2), and metoprolol has a closest similar decreasing HR effect on heart rate with acebutolol [15]. Here, we performed a retrospective study to compare the blood pressure changes taken metoprolol between male and female from two Chinese northern communities. In a diabetes cohort from Beijing city, with a total of 35 patients who receiving metoprolol were recruited (basal clinical data see Table 1), female patients exhibited lower DBP than male patients (Table 1, P = 0.001) although with larger BMI (P = 0.035). Our data showed that metoprolol treatment had more anti-hypertension effect in obese females than lean males, these differences showed the stronger evidence for sex-response of metoprolol to support our in silico model. In the second cohort with 153 patients (86 males vs. 67 female patients) who were from the Jidong community at Tangshan city receiving metoprolol, the DBP decrease by the drug was more prominent in the female than male patients (Table 2, P = 0.0004). Together, these data demonstrate the accuracy and clinical relevance of our prediction model.

## Discussion

Using accumulating big datasets in public databases, we have developed a computational model for the prediction of drugs exerting sex-specific effects. Dissecting the differences between the sexes in health and disease is crucial for the improvement of the efficiency, efficacy, accuracy and precision of medical care. However, this has not received much attention in both medical care and clinical research. Thousands of drugs have been approved [14], but systematic evaluation for sex-specific effects has been lacking. Computational approaches and frameworks for these purposes are still not readily available. The model presented here addresses this major gap. The accuracy of this model was confirmed by animal experimentation with two positive predictions for sex-specific effects, i.e. acebutolol and tacrine, and one negative prediction for sex-specific effects, i.e. metformin. Importantly, the clinical relevance of our model was demonstrated in studies with male and female patients receiving metoprolol, which showed that a significantly lower DBP was achieved in female than male patients.

In fact, in our attempt to validate the model in humans, we tried to employ publicly available clinical data to support the predictions. Sutandar et al. [16] reported the acebutolol plus hydrochlorothiazide effects for the treatment of essential hypertension. Since detailed data were provided in the study for 11 male and 11 female patients with acebutolol treatment, we performed a meta-analysis for any differences between men and women, but we did not find significant effects in SBP (Additional file 5: Figure S3a), DBP (Additional file 5: Figure S3b) or HR (Additional file 5: Figure S3c). Analysis of absolute heart rate values revealed significant effects of acebutolol in both sexes. However, this study did not exclude the interference of combination with other drugs, such as diuretics, and the sample size was rather to small significant sex differences. To this extent, in multicenter clinical trials with acebutolol alone for the treatment of essential hypertension, SBP and DBP were lowered effectively at rest but not exercise [17–20]. Another multicenter clinical trial reported significant effects of acebutolol and confirming its anti-hypertensive actions [21]. However, none of these studies compared the responses to acebutolol between men and women. For our clinical validation, we chose metoprolol, due to its similar structure to acebutolol and that its use has been limited in China because of its side-effects. In first cohort, obese female patients got the more lowering blood pressure effect than lean male patients (overweight and obesity is a risk factor for cardiovascular diseases and diabetes), the great differences supported the sex-response of metoprolol. The second cohort’s study also showed the same sex-response of metoprolol in age, gender, BMI and biochemical parameters matched patients. Our data show for the first time that the response to this drug differs significantly between men and women, which is also a strong clinical confirmation for our model.

Our study was limited by scarce sex-specific genomic and transcriptomic data, especially, data on sex-specific responses to first-line drugs. This lack of data limited our prediction model in assessing sex-specific responses to further drugs. Another limitation is that gene expression profile based approaches do not address direct protein targets of the interested drugs. The expression profile-derived “gene signature” represent one type of “molecular phenotype” but not the drug targets. Although this limitation existed, this class of approaches has been successfully applied in a number of studies in recent years. In addition, genetic or genomic variations in drug target DNA regions could lead to changing in drug effects. Therefore, integrating GWAS data and eQTL data into this study could be useful to improve the prediction accuracy.

## Conclusion

In summary, we developed an in silico model for the prediction of drugs exerting sex-specific effects. On the basis of this, possible responses or therapeutic effects of drugs to male and female patients using our prediction model may easily be evaluated, to avoid side-effects or adjust treatments accordingly. We put forward that our model may contribute to more appropriate and personalized pharmacological intervention approaches.

## Additional files

**Additional file 1.** List of female-biased genes and male biased genes on human heart.

**Additional file 2.** List of predicted sex-responsive drugs on human heart.

**Additional file 3: Figure S1.** The mean DHE fluorescence density analysis from DHE staining.

**Additional file 4: Figure S2.** The chemical structures of acebutolol and metoprolol. The red circle showed the difference of two drugs in chemical groups.

**Additional file 5: Figure S3.** The changes of hemodynamic changes after administration of acebutolol combination with hydrochlorothiazide. The systolic blood pressure (a), diastolic blood pressure (b), the minus of heart rate (c) and heart rate (d) changes after taken acebutolol combination and hydrochlorothiazide for 12 weeks from study of Sutandar.
