# Supplementary material for: Large-scale in silico identification of drugs exerting sex-specific effects in the heart
Source: J Transl Med. 2018 Aug 29;16:236. doi: 10.1186/s12967-018-1612-6 (PMC6116388; doi:10.1186/s12967-018-1612-6)
Supplement: Supplementary file 1 — Additional file 1. List of female-biased genes and male biased genes on human heart. [file 12967_2018_1612_MOESM1_ESM.docx]

**Additional File 1. List of predicted sex-responsive drugs on human heart.**

| Drug | OR | p-value |
| --- | --- | --- |
| Tacrine | 19.5 | 0.001953306 |
| tolnaftate | 34.66667 | 0.002137569 |
| retrorsine | 16.25 | 0.002509272 |
| H-89 | 33 | 0.002759456 |
| colecalciferol | 0.05555556 | 0.003888266 |
| trazodone | 35 | 0.004524887 |
| hydrocortisone | 50 | 0.005413704 |
| androsterone | 12.44444 | 0.006158279 |
| phthalylsulfathiazole | 25.66667 | 0.00619195 |
| PHA-00846566E | 30 | 0.006271334 |
| pentamidine | 13.5 | 0.007234844 |
| moroxydine | 23.33333 | 0.007518797 |
| etamsylate | 0.05454545 | 0.008244455 |
| acebutolol | 27 | 0.01282051 |
| aciclovir | 40 | 0.01282051 |
| econazole | 40 | 0.01282051 |
| haloperidol | 16 | 0.01305694 |
| CAY-10397 | 12.375 | 0.0154193 |
| kanamycin | 9.8 | 0.016595 |
| 6-benzylaminopurine | 15.42857 | 0.01685086 |
