# Supplementary material for: Large-scale in silico identification of drugs exerting sex-specific effects in the heart
Source: J Transl Med. 2018 Aug 29;16:236. doi: 10.1186/s12967-018-1612-6 (PMC6116388; doi:10.1186/s12967-018-1612-6)
Supplement: Supplementary file 2 — Additional file 2. List of predicted sex-responsive drugs on human heart. [file 12967_2018_1612_MOESM2_ESM.docx]

**Additional File 2. List of female-biased genes and male biased genes on human heart.**

| gene | chr | stand | from | to | fold | p-value | FDR | Sex |
| --- | --- | --- | --- | --- | --- | --- | --- | --- |
| NCRNA00185 | chrY | - | 21034391 | 21040114 | 3.6 | 1.51E-48 | 3.09E-44 | Male |
| RPS4Y1 | chrY | + | 2709527 | 2734997 | 9.5 | 2.53E-48 | 3.09E-44 | Male |
| KDM5D | chrY | - | 21867301 | 21906824 | 7.3 | 2.78E-48 | 3.09E-44 | Male |
| CYorf15B | chrY | + | 21754336 | 21768160 | 3.6 | 2.68E-46 | 2.09E-42 | Male |
| TTTY14 | chrY | - | 21094585 | 21239302 | 3.7 | 3.14E-46 | 2.09E-42 | Male |
| DDX3Y | chrY | + | 15016019 | 15032390 | 9.9 | 1.65E-45 | 9.16E-42 | Male |
| USP9Y | chrY | + | 14813160 | 14972957 | 8.1 | 2.41E-45 | 1.08E-41 | Male |
| UTY | chrY | - | 15360259 | 15592553 | 9.4 | 2.59E-45 | 1.08E-41 | Male |
| CYorf15A | chrY | + | 21729236 | 21752308 | 5.5 | 5.97E-45 | 2.21E-41 | Male |
| PRKY | chrY | + | 7142013 | 7249589 | 2.7 | 9.38E-45 | 3.12E-41 | Male |
| CD24 | chrY | - | 21152510 | 21154705 | 5.1 | 1.10E-44 | 3.33E-41 | Male |
| ZFY | chrY | + | 2803322 | 2850547 | 3.4 | 6.12E-43 | 1.70E-39 | Male |
| EIF1AY | chrY | + | 22737611 | 22755409 | 14.7 | 8.20E-43 | 2.10E-39 | Male |
| TTTY10 | chrY | - | 22627554 | 22681114 | 4.8 | 4.82E-42 | 1.15E-38 | Male |
| ZFX | chrX | + | 24167729 | 24234206 | 0.7 | 1.37E-30 | 3.04E-27 | Female |
| RPS4Y2 | chrY | + | 22917954 | 22942918 | 1.3 | 2.94E-27 | 5.76E-24 | Male |
| KDM6A | chrX | + | 44732757 | 44971847 | 0.7 | 2.39E-22 | 3.79E-19 | Female |
| MBNL3 | chrX | - | 131506029 | 131623996 | 2.7 | 1.61E-20 | 2.44E-17 | Male |
| CD99 | chrX | + | 2609220 | 2659350 | 1.3 | 2.24E-20 | 3.11E-17 | Male |
| CD99 | chrY | + | 2559225 | 2609350 | 1.3 | 2.24E-20 | 3.11E-17 | Male |
| STS | chrX | + | 7137497 | 7272851 | 0.8 | 1.33E-16 | 1.77E-13 | Female |
| KDM5C | chrX | - | 53220653 | 53254604 | 0.8 | 1.26E-15 | 1.61E-12 | Female |
| NLGN4Y | chrY | + | 16634632 | 16957530 | 1.4 | 1.50E-14 | 1.85E-11 | Male |
| FMO5 | chr1 | - | 146657838 | 146696922 | 2.5 | 2.09E-14 | 2.49E-11 | Male |
| SMC1A | chrX | - | 53401070 | 53449618 | 0.8 | 5.01E-13 | 5.62E-10 | Female |
| FRMD5 | chr15 | - | 44162962 | 44487429 | 1.3 | 5.06E-13 | 5.62E-10 | Male |
| NCRNA00183 | chrX | + | 73219444 | 73220142 | 0.8 | 3.27E-12 | 3.51E-09 | Female |
| FAM83B | chr6 | + | 54711564 | 54806883 | 2.0 | 7.98E-12 | 8.30E-09 | Male |
| TXLNG | chrX | + | 16804550 | 16862642 | 0.8 | 5.74E-11 | 5.79E-08 | Female |
| FAM155B | chrX | + | 68725078 | 68752351 | 1.8 | 9.58E-10 | 9.38E-07 | Male |
| DDX3X | chrX | + | 41192651 | 41223725 | 0.8 | 1.05E-09 | 9.99E-07 | Female |
| ASMTL | chrX | - | 1522032 | 1571822 | 1.2 | 2.56E-09 | 2.32E-06 | Male |
| ZBED1 | chrY | - | 2087547 | 2369008 | 1.1 | 2.65E-09 | 2.32E-06 | Male |
| ZBED1 | chrX | - | 2137547 | 2419008 | 1.1 | 2.65E-09 | 2.32E-06 | Male |
| CLIC2 | chrX | - | 154505500 | 154563986 | 0.7 | 4.29E-09 | 3.66E-06 | Female |
| ASMTL | chrY | - | 1472032 | 1521756 | 1.2 | 5.86E-09 | 4.88E-06 | Male |
| CA5B | chrX | + | 15756392 | 15805748 | 0.8 | 3.26E-08 | 2.65E-05 | Female |
| HDHD1 | chrX | - | 6966916 | 7066223 | 0.9 | 4.43E-08 | 3.51E-05 | Female |
| EIF2S3 | chrX | + | 24073065 | 24096927 | 0.9 | 4.53E-08 | 3.51E-05 | Female |
| ZNF502 | chr3 | + | 44754138 | 44765323 | 0.9 | 4.69E-08 | 3.55E-05 | Female |
| SLC15A2 | chr3 | + | 121613287 | 121660458 | 1.5 | 6.92E-08 | 5.12E-05 | Male |
| SV2B | chr15 | + | 91643545 | 91838650 | 1.8 | 7.30E-08 | 5.28E-05 | Male |
| TMSB4Y | chrY | + | 15815447 | 15817904 | 1.1 | 9.86E-08 | 6.99E-05 | Male |
| MDGA1 | chr6 | - | 37600284 | 37665766 | 1.3 | 2.22E-07 | 1.49E-04 | Male |
| DOK5 | chr20 | + | 53092163 | 53267710 | 0.6 | 2.23E-07 | 1.49E-04 | Female |
| SYAP1 | chrX | + | 16737755 | 16783459 | 0.9 | 4.43E-07 | 2.89E-04 | Female |
| HS6ST3 | chr13 | + | 96743001 | 97491812 | 1.5 | 4.95E-07 | 3.17E-04 | Male |
| SAMD5 | chr6 | + | 147830063 | 147891157 | 0.8 | 6.58E-07 | 4.13E-04 | Female |
| RAPGEF6 | chr5 | - | 130761582 | 130970929 | 0.9 | 1.17E-06 | 7.08E-04 | Female |
| BMI1 | chr10 | + | 22610140 | 22620413 | 0.9 | 1.30E-06 | 7.73E-04 | Female |
| CACNA2D3 | chr3 | + | 54156689 | 55108584 | 1.4 | 1.58E-06 | 9.23E-04 | Male |
| FABP6 | chr5 | + | 159614374 | 159665729 | 1.3 | 2.04E-06 | 1.17E-03 | Male |
| SCARNA9L | chrX | - | 20154184 | 20154253 | 0.7 | 2.80E-06 | 1.58E-03 | Female |
| BTBD10 | chr11 | - | 13409555 | 13484838 | 0.9 | 3.58E-06 | 1.99E-03 | Female |
| NPTX2 | chr7 | + | 98246604 | 98259181 | 1.3 | 4.21E-06 | 2.30E-03 | Male |
| WRN | chr8 | + | 30890778 | 31031277 | 0.9 | 4.60E-06 | 2.45E-03 | Female |
| ATP6V0E2 | chr7 | + | 149570054 | 149577787 | 1.2 | 4.64E-06 | 2.45E-03 | Male |
| JMJD1C | chr10 | - | 64926981 | 65028982 | 0.9 | 4.74E-06 | 2.47E-03 | Female |
| ZNF470 | chr19 | + | 57079377 | 57090119 | 0.9 | 4.89E-06 | 2.50E-03 | Female |
| CPLX3 | chr15 | + | 75117817 | 75119208 | 1.3 | 7.83E-06 | 3.95E-03 | Male |
| FRAS1 | chr4 | + | 78979096 | 79465423 | 0.8 | 1.20E-05 | 5.96E-03 | Female |
| DNER | chr2 | - | 230222345 | 230579286 | 1.3 | 1.32E-05 | 6.33E-03 | Male |
| STARD4 | chr5 | - | 110831731 | 110848621 | 0.8 | 1.32E-05 | 6.33E-03 | Female |
| PRDM5 | chr4 | - | 121615929 | 121844025 | 0.9 | 1.33E-05 | 6.33E-03 | Female |
| TPP1 | chr11 | - | 6634000 | 6640660 | 1.1 | 1.43E-05 | 6.71E-03 | Male |
| GTPBP6 | chrX | - | 220025 | 230886 | 1.1 | 1.50E-05 | 6.93E-03 | Male |
| CPLX3 | chr15 | + | 75118947 | 75124141 | 1.4 | 1.52E-05 | 6.93E-03 | Male |
| RNF187 | chr1 | + | 228675068 | 228683467 | 1.1 | 1.57E-05 | 7.06E-03 | Male |
| PGAP2 | chr11 | + | 3819143 | 3847584 | 1.3 | 1.69E-05 | 7.40E-03 | Male |
| TMEM161A | chr19 | - | 19230426 | 19249287 | 1.1 | 1.69E-05 | 7.40E-03 | Male |
| TEC | chr4 | - | 48137800 | 48271881 | 0.9 | 1.78E-05 | 7.64E-03 | Female |
| HIST1H2BK | chr6 | - | 27106072 | 27114627 | 0.8 | 1.79E-05 | 7.64E-03 | Female |
| RTN2 | chr19 | - | 45988108 | 46000313 | 1.1 | 1.86E-05 | 7.84E-03 | Male |
| HOPX | chr4 | - | 57514164 | 57547870 | 1.3 | 2.04E-05 | 8.43E-03 | Male |
| CDC73 | chr1 | + | 193090919 | 193223031 | 0.9 | 2.05E-05 | 8.43E-03 | Female |
| MPI | chr15 | + | 75182372 | 75191774 | 1.3 | 2.29E-05 | 9.30E-03 | Male |
| TRIM44 | chr11 | + | 35684353 | 35829775 | 0.9 | 2.49E-05 | 9.99E-03 | Female |
| TMEM143 | chr19 | - | 48835613 | 48867208 | 1.2 | 2.53E-05 | 1.00E-02 | Male |
| ZC3H12B | chrX | + | 64708706 | 64727767 | 0.9 | 2.73E-05 | 1.07E-02 | Female |
| ASIP | chr20 | + | 32848171 | 32857606 | 1.3 | 2.83E-05 | 1.10E-02 | Male |
| POC5 | chr5 | - | 74969949 | 75013233 | 0.9 | 3.25E-05 | 1.23E-02 | Female |
| FAM188A | chr10 | - | 15820169 | 15902519 | 0.9 | 3.54E-05 | 1.32E-02 | Female |
| PPM1E | chr17 | + | 56833232 | 57062537 | 1.3 | 3.86E-05 | 1.43E-02 | Male |
| CSRP2 | chr12 | - | 77252005 | 77272840 | 1.2 | 4.17E-05 | 1.53E-02 | Male |
| SR140 | chr3 | + | 142720010 | 142776492 | 0.9 | 4.27E-05 | 1.55E-02 | Female |
| GNAL | chr18 | + | 11689263 | 11881921 | 1.3 | 4.68E-05 | 1.67E-02 | Male |
| PCDH20 | chr13 | - | 61983818 | 61989396 | 0.5 | 4.71E-05 | 1.67E-02 | Female |
| SIPA1L1 | chr14 | + | 71996032 | 72206122 | 0.9 | 4.93E-05 | 1.71E-02 | Female |
| SNORA40 | chr2 | + | 135894198 | 135894327 | 0.8 | 4.93E-05 | 1.71E-02 | Female |
| ADAM11 | chr17 | + | 42836568 | 42859214 | 1.3 | 5.06E-05 | 1.74E-02 | Male |
| GRID1 | chr10 | - | 87359312 | 88126283 | 0.9 | 5.26E-05 | 1.77E-02 | Female |
| SP3 | chr2 | - | 174773258 | 174830430 | 0.9 | 5.78E-05 | 1.92E-02 | Female |
| STAG1 | chr3 | - | 136055076 | 136471245 | 0.9 | 6.06E-05 | 1.99E-02 | Female |
| ITPKA | chr15 | + | 41786073 | 41795749 | 1.1 | 6.57E-05 | 2.12E-02 | Male |
| CAMTA1 | chr1 | + | 6845384 | 7829764 | 0.9 | 6.61E-05 | 2.12E-02 | Female |
| QSER1 | chr11 | + | 32914792 | 33005773 | 0.9 | 6.88E-05 | 2.18E-02 | Female |
| NAA10 | chrX | - | 153194695 | 153200531 | 0.9 | 6.99E-05 | 2.20E-02 | Female |
| ELAVL3 | chr19 | - | 11562143 | 11591808 | 1.1 | 7.22E-05 | 2.25E-02 | Male |
| ARL13B | chr3 | + | 93698982 | 93774123 | 0.9 | 7.79E-05 | 2.40E-02 | Female |
| OVOS2 | chr12 | - | 31264593 | 31354168 | 1.1 | 8.27E-05 | 2.53E-02 | Male |
| MYEF2 | chr15 | - | 48432427 | 48470513 | 0.8 | 8.79E-05 | 2.66E-02 | Female |
| NIPAL3 | chr1 | + | 24742293 | 24799466 | 1.2 | 8.95E-05 | 2.68E-02 | Male |
| GPX3 | chr5 | + | 150399919 | 150409051 | 1.1 | 1.04E-04 | 3.11E-02 | Male |
| HMGCL | chr1 | - | 24128322 | 24152677 | 1.2 | 1.07E-04 | 3.13E-02 | Male |
| CLPP | chr19 | + | 6361463 | 6368915 | 1.1 | 1.08E-04 | 3.13E-02 | Male |
| SLC36A2 | chr5 | - | 150694539 | 150727120 | 1.2 | 1.11E-04 | 3.17E-02 | Male |
| C10orf28 | chr10 | + | 99894428 | 100004654 | 0.9 | 1.11E-04 | 3.17E-02 | Female |
| DHX15 | chr4 | - | 24529087 | 24586185 | 0.9 | 1.13E-04 | 3.18E-02 | Female |
| ETNK2 | chr1 | - | 204100190 | 204121131 | 1.5 | 1.18E-04 | 3.29E-02 | Male |
| ZNF410 | chr14 | + | 74353508 | 74398981 | 0.9 | 1.19E-04 | 3.29E-02 | Female |
| TUBB4 | chr19 | - | 6494330 | 6502330 | 1.2 | 1.20E-04 | 3.30E-02 | Male |
| RPS4X | chrX | - | 71491892 | 71497150 | 0.9 | 1.26E-04 | 3.42E-02 | Female |
| MCTP2 | chr15 | + | 94774951 | 95023633 | 0.6 | 1.32E-04 | 3.55E-02 | Female |
| ASTN2 | chr9 | - | 119187507 | 120177317 | 1.1 | 1.36E-04 | 3.63E-02 | Male |
| CCDC99 | chr5 | + | 169010659 | 169031782 | 0.9 | 1.40E-04 | 3.71E-02 | Female |
| MIR323 | chr14 | + | 101492069 | 101492154 | 1.1 | 1.44E-04 | 3.76E-02 | Male |
| FRG1B | chr20 | + | 29611857 | 29652264 | 1.2 | 1.48E-04 | 3.83E-02 | Male |
| DAZ1 | chrY | + | 26979980 | 27053187 | 1.1 | 1.61E-04 | 4.02E-02 | Male |
| DAZ1 | chrY | - | 25275502 | 25345241 | 1.1 | 1.61E-04 | 4.02E-02 | Male |
| DAZ2 | chrY | + | 25365594 | 25437503 | 1.1 | 1.61E-04 | 4.02E-02 | Male |
| WSCD1 | chr17 | + | 5973934 | 6027747 | 1.1 | 1.61E-04 | 4.02E-02 | Male |
| NIT1 | chr1 | + | 161087872 | 161095235 | 1.1 | 1.63E-04 | 4.05E-02 | Male |
| C19orf39 | chr19 | + | 11485361 | 11487627 | 1.1 | 1.68E-04 | 4.14E-02 | Male |
| ATP5SL | chr19 | - | 41937223 | 41945874 | 1.1 | 1.76E-04 | 4.26E-02 | Male |
| KCNJ3 | chr2 | + | 155555093 | 155713014 | 1.5 | 1.76E-04 | 4.26E-02 | Male |
| EIF1AX | chrX | - | 20142636 | 20159966 | 0.8 | 1.76E-04 | 4.26E-02 | Female |
| PTPRA | chr20 | + | 2821366 | 3019320 | 1.0 | 1.78E-04 | 4.26E-02 | Female |
| ACPL2 | chr3 | + | 140950682 | 141013745 | 0.7 | 1.79E-04 | 4.26E-02 | Female |
| ESR2 | chr14 | - | 64550944 | 64805317 | 1.1 | 1.87E-04 | 4.41E-02 | Male |
| ZNF131 | chr5 | + | 43121642 | 43176116 | 0.9 | 1.93E-04 | 4.50E-02 | Female |
| FHIT | chr3 | - | 59735036 | 61237124 | 1.2 | 1.96E-04 | 4.51E-02 | Male |
| NCSTN | chr1 | + | 160313054 | 160328742 | 1.1 | 1.97E-04 | 4.51E-02 | Male |
| GSTM2 | chr1 | - | 149278785 | 149278857 | 1.1 | 2.09E-04 | 4.72E-02 | Male |
| NGFRAP1 | chrX | + | 102631254 | 102633005 | 1.2 | 2.17E-04 | 4.87E-02 | Male |
