# Supplementary figures and images for: Large-scale in silico identification of drugs exerting sex-specific effects in the heart
Source: J Transl Med. 2018 Aug 29;16:236. doi: 10.1186/s12967-018-1612-6 (PMC6116388; doi:10.1186/s12967-018-1612-6)

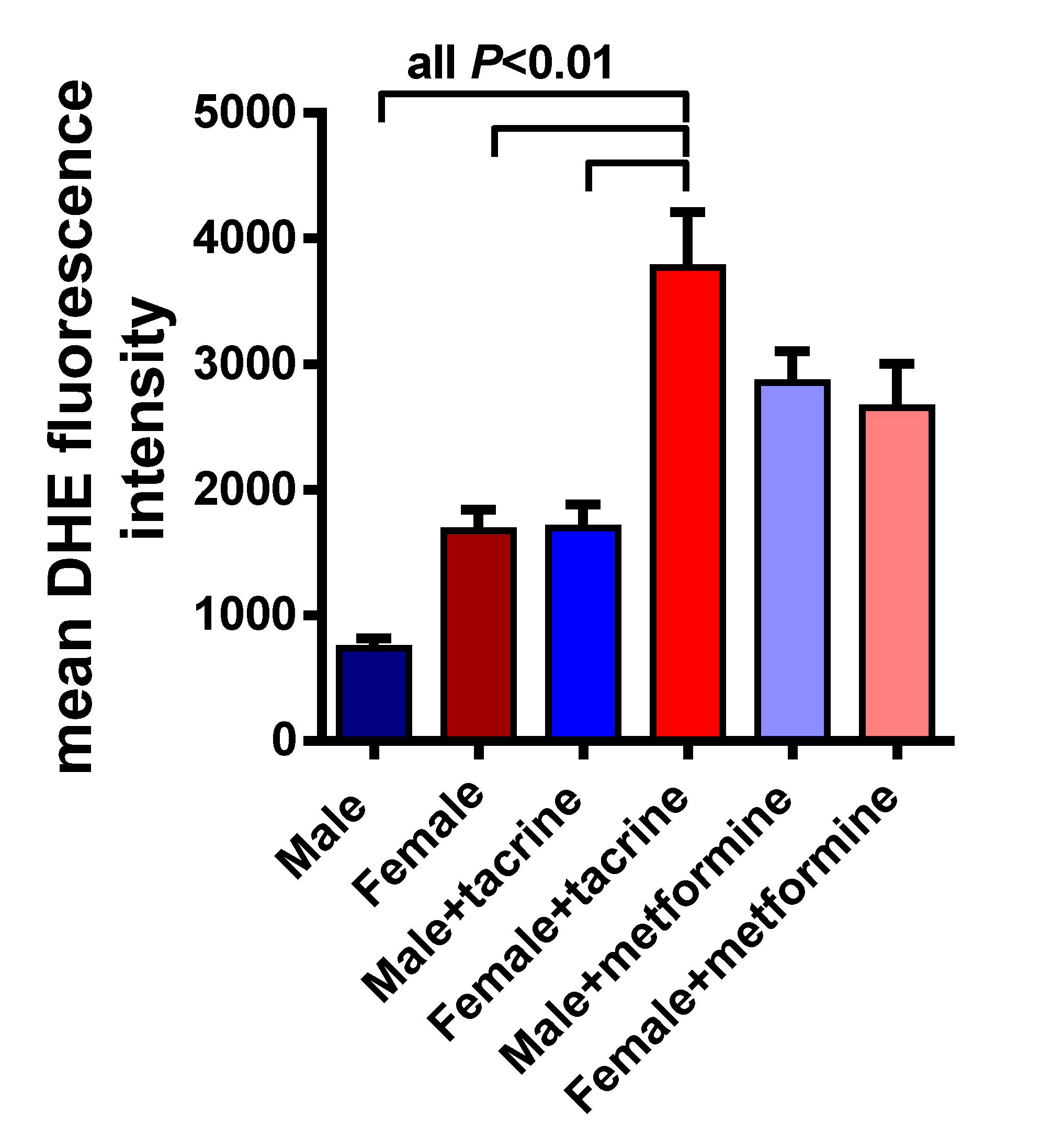

Supplement: Supplementary file 3 — Additional file 3: Figure S1. The mean DHE fluorescence density analysis from DHE staining. [file 12967_2018_1612_MOESM3_ESM.tiff]

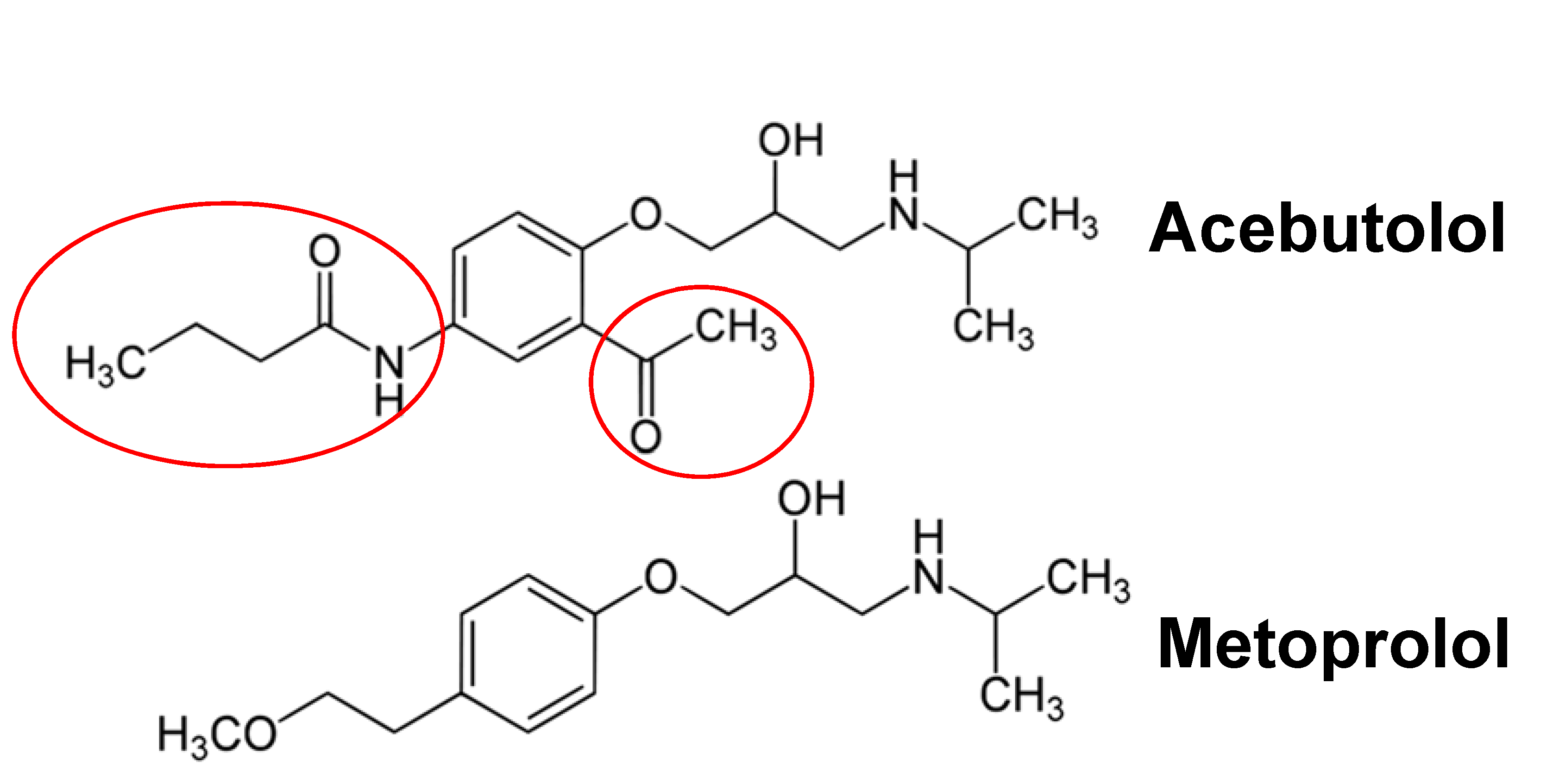

Supplement: Supplementary file 4 — Additional file 4: Figure S2. The chemical structures of acebutolol and metoprolol. The red circle showed the difference of two drugs in chemical groups. [file 12967_2018_1612_MOESM4_ESM.tiff]

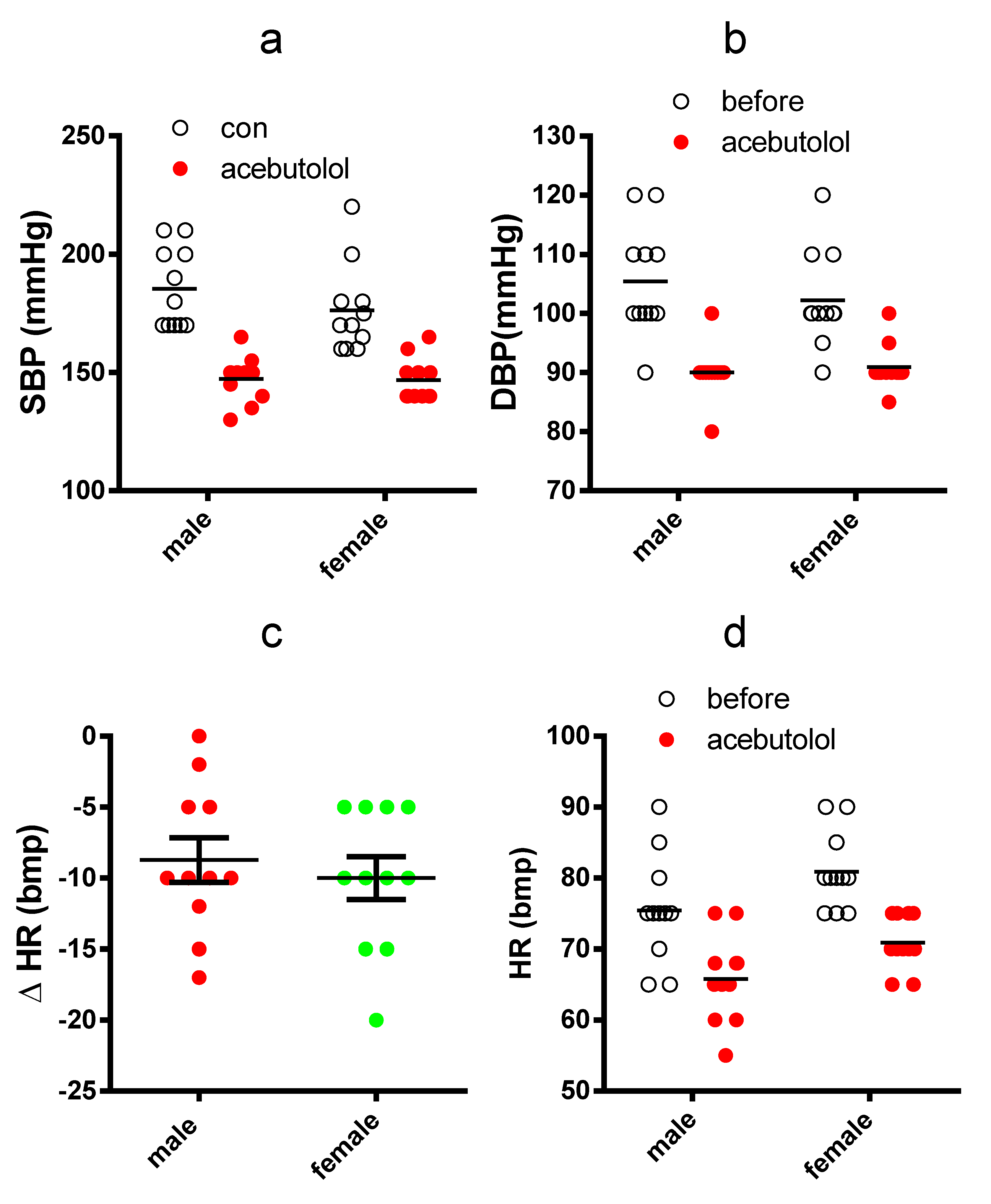

Supplement: Supplementary file 5 — Additional file 5: Figure S3. The changes of hemodynamic changes after administration of acebutolol combination with hydrochlorothiazide. The systolic blood pressure (a), diastolic blood pressure (b), the minus of heart rate (c) and heart rate (d) changes after taken acebutolol combination and hydrochlorothiazide for 12 weeks from study of Sutandar. [file 12967_2018_1612_MOESM5_ESM.tiff]
